# Comparison of the outcomes of laparoscopic pyeloplasty with and without concomitant pyelolithotomy

**DOI:** 10.1590/S1677-5538.IBJU.2018.0781

**Published:** 2019-01-29

**Authors:** Mustafa Kadihasanoglu, Ugur Yucetas, Emre Karabay, Erkan Sönmezay

**Affiliations:** 1 Department of Urology Istanbul Training & Research Hospital Istanbul Turkey Department of Urology, Istanbul Training & Research Hospital, Istanbul, Turkey

**Keywords:** Laparoscopy, Pyeloform [Supplementary Concept], Cakut [Supplementary Concept]

## Abstract

**Objective:**

We aimed to evaluate the results of laparoscopic pyeloplasty with concomitant pyelolithotomy and compare results with a cohort of patients undergoing laparoscopic pyeloplasty without pyelolithotomy.

**Materials and Methods:**

We retrospectively reviewed records of 43 patients undergoing transperitoneal laparoscopic Anderson-Hynes dismembered pyeloplasty between December 2012 and July 2018 at our department. Eighteen patients (42%) underwent laparoscopic pyeloplasty with concomitant pyelolithotomy. The results of patients with renal stones were compared with 25 matched patients undergoing laparoscopic pyeloplasty without concomitant renal stones. Demographic data, operative and stone parameters were compared between the groups.

**Results:**

The groups were similar regarding to demographic characteristics. All operations were completed laparoscopically with no conversions to open surgery. In 3 cases without renal stones and 15 cases with renal stones, transposition of the ureter due to crossing vessels was performed. The mean stone size was 13±5.24 mm, and the median number of stones was 1 (1-18). The success of laparoscopic pyeloplasty with and without pyelolithotomy was 93.3% and 92.9%, respectively, as confirmed by negative diuretic renogram at postoperative 3rd months. Overall stone-free rate after laparoscopic pyelolithotomy was 93.3%. Mean operative time was 222.6765.71 minutes vs. 219.11±75.63 minutes for the pyeloplasty with concomitant pyelolithotomy vs. pyeloplasty, respectively (p=0.88).

**Conclusions:**

Laparoscopic pyeloplasty with concomitant pyelolithotomy is a safe and effective intervention with associated good cosmetic results and high stone-free rates without significant increase in operative time or complications.

## INTRODUCTION

Ureteropelvic junction (UPJ) obstruction is the most frequently seen congenital abnormality of the upper urinary tract, with an incidence of 1 per 500 live births screened by routine antenatal ultrasound. A 70-fold increased risk of developing renal stones in UPJ obstruction has been estimated by Husmann et al. (1), due to stasis and infection associated with upper urinary tract obstruction.

The coexistence of UPJ obstruction and renal stone may worsen symptoms such as pain and fever, throughout the course of upper and lower urinary tract infections. The presence of nephrolithiasis in patients with UPJ obstruction is an unquestionable indication for pyeloplasty with pyelolithotomy. Urologists face a therapeutic dilemma in patients with renal stones and UPJ obstruction, because of the selection of the appropriate treatment, because the extraction of stones from the renal pelvis is suitable at the time of dismembering the ureter from the pelvis; however, calyceal stones in peripheral calyxes with tight ostia are not very easy to treat. Although concurrent performance of open pyeloplasty and pyelolithotomy remains the gold standard therapy for the patients with UPJ obstruction and renal stones, multiple minimally invasive methods have been used for the surgical management of this coexistence. Laparoscopic transperitoneal pyeloplasty, which was introduced by Schuessler et al. in 1993 to minimize the morbidity associated with open pyeloplasty, is a preferred technique to open procedure for the outcomes (2). Recently, percutaneous endopyelotomy with nephrolithotomy, and laparoscopic pyeloplasty with concomitant pyelolithotomy are the preferred minimally invasive procedures for the coexistence of UPJ obstruction and nephrolithiasis (3, 4). The aim of this study was to compare the outcomes of laparoscopic pyeloplasty with and without pyelolithotomy.

## MATERIALS AND METHODS

Between December 2012 and July 2018, 43 patients (43 renal units) with symptomatic UPJ obstruction that underwent laparoscopic repair and completed at least 3 months of postoperative follow-up were included in this retrospective study. Fifteen of these patients had concomitant non-obstructing pelvic or calyceal stones. We evaluated 25 men and 18 women with a mean age 33.63±12.7 years (range, 11-57 years). They were symptomatic prior to diagnosis, and presented with flank pain on the side of stone. Stones were on right side in 11, on the left side in 4 patients. Many patients had associated symptoms, such as hematuria or recurrent urinary tract infection. The patients were divided in two groups based on surgical techniques applied as follows: only laparoscopic pyeloplasty (Group-1; n=25), laparoscopic pyeloplasty with concomitant pyelolithotomy (Group-2; n=18).

The preoperative diagnosis of the UPJ obstruction, and the number, size and location of stones were determined by a combination of helical computerized tomography (CT) with and without contrast, intravenous pyelography (IVP), and diethylenetriaminepentaacetic acid (DTPA) diuretic renogram. Obstruction was defined as half-time more than 20 minutes after diuretic on renal scan, and as delayed nephrogram and/or excretion with hydronephrosis on radiological examinations. Indication of operation was made based on the presence of obstruction, clinical presentation of patient, and radiological findings. None of our patients had a history of previous surgical procedures for UPJ obstruction. All of patients were first-time stone formers with no history of stones or stone-related operations. The patients were subjected to transperitoneal laparoscopic Hynes-Anderson dismembered pyeloplasty and, if indicated, concomitant pyelolithotomy. No patients had intrarenal lithotripsy procedures.

The patient’s charts were reviewed retrospectively to analyze grade of preoperative hydronephrosis (according to Society of Fetal Urology), number and size of stones, presence of crossing vessels, the duration of hospital stay, estimated blood loss, operative time, and anastomosis time. The operative time was defined as the time from first skin incision to last skin suture.

After surgery, patients were evaluated with a kidney-ureter-bladder film and renal ultrasound before discharge to check for residual stones. All patients were evaluated with DTPA scan and IVP after 3 months of surgery, and with ultrasound and DTPA scan annually. Success was defined as resolution of preoperative symptoms and hydronephrosis on diuretic renogram. Intraoperative complications were classified according to the Satava classification system (5), and postoperative complications were grouped based on the Clavien-Dindo grading system (6).

The pneumoperitoneum was established with a Veress needle positioned in the umbilicus and maintained at 12-15mm Hg throughout the procedure after induction of general anesthesia. The patient was placed in a modified 45º lateral decubitus position with the affected side up. After the abdomen was insufflated, a standard three-port transperitoneal technique was performed to maximize the working space and anatomical orientation. An 11mm trocar was placed in umbilicus for the camera in slim patients and children. In regular patients, 11mm trocar was placed lateral and superior to the umbilicus at the lateral border of the rectus abdominalis muscle. Five and 12mm trocars were placed at the midclavicular line, at the spino-umbilical line and subcostally, respectively. An additional 5mm port was used if needed.

After achieving pneumoperitoneum, the peritoneal incision along the white line of Toldt, division of the renocolic ligament, and the reflection of the colon medially off the kidney were performed using standard laparoscopic techniques in order to provide clear exposure of the ureter and renal pelvis. After sharp and blunt dissection of the renal pelvis, and making it free from adjacent structures, an initial pyelotomy incision was performed, in order to remove all renal and calyceal stones. The initial incision for mini-pyelotomy was made with the decision to perform Anderson-Hynes dismembered pyeloplasty, because this incision was incorporated into the final pyeloplasty. This incision was just long enough to use laparoscopic grasper of flexible cystoscope. The extraction of stones was accomplished by using laparoscopic graspers, and irrigation for flushing out smaller stones in the calices. If the stones could not be removed by these techniques, flexible cystoscope was placed through 12mm trocar with irrigation, and stone extraction was performed under direct vision using nitinol basket. Intraoperative fluoroscopy was used to confirm the clearance of stones. After the pyelolithotomy, by extending initial pyelotomy, redundant pelvic tissue was removed, the UPJ was circumferentially transected, and the ureter was spatulated at the lateral border towards the lower pole of the kidney to a sufficient length. In case of a crossing vessel, the same technique was used, and prior to the initiation of the anastomosis the ureter was transposed to the anterior of the vessel. A classic Anderson-Hynes pyeloplasty was performed using two running 4-0 polyglycolic-acid sutures for both anterior and posterior anastomosis. Intracorporeal knot tying was performed in a free-hand fashion. After the completion of the posterior wall suturing, a guidewire was inserted through a trocar in the ureter reaching the bladder, and a 4.7F double J (DJ) stent was placed in an antegrade fashion. After completing the anastomosis, a drain was finally passed through one of the port side in the retroperitoneum posterior to the anastomosis.

The urethral catheter was removed on first day of operation and the drain was removed when drain output was less than 50mL, after which was removed and the patient was discharged. The DJ stent was removed by cystoscopy after 6 weeks.

For statistical analyses, SPSS 14 (SPSS Inc., Chicago, IL, USA) was used. All data were expressed as the mean±standard deviation or median (interquartile range). The distribution of continuous variables was evaluated according to the Shapiro-Wilk normality test. If the distribution was normal, Student-t test was used for statistical analysis; if the distribution was not normal, Mann-Whitney U test was used. The categorical variables were analyzed by Fisher-exact test (two-tailed) or chi-square. A P value less than 0.05 was considered statistically significant.

## RESULTS

The patients with renal stones were older than the patients without kidney stone (26.9410.94 vs. 41.07±12.73 years, p=0.004). The groups were similar regarding to other demographic characteristics and preoperative renal function (Table-1). In 15 patients of group-1, and 3 patients of group-2, transposition of the ureter was performed due to crossing vessel (p=0.03).

**Table 1:** Baseline demographics and clinical characteristics.

	Group 1 (n=28)	Group 2 (n=15)	p
Age [years, mean±SD, (range)]	29.64±10.94 (11-53)	41.07±12.73 (24-57)	**0.004**
Gender [male/female, n (%)]	15 (53.6) / 13 (46.4)	10 (66.7) / 5 (33.3)	0.41
BMI [kg/m^2^, mean±SD, (range)]	24.37±5.23 (18.03-4297)	27.40±5.38 (23.46-40.89)	0.15
Side [L/R, n (%)]	9 (32.1)/19 (67.9)	4 (26.7)/11 (73.3)	0.71
**Grade of hydronephrosis [n (%)]**			**0.69**
Grade 1	0 (0)	0 (0)	
Grade 2	12 (42.9)	6 (40)	
Grade 3	12 (42.9)	8 (53.3)	
Grade 4	4 (14.2)	1 (6.7)	
Crossing vessel [n (%)]	15 (53.6)	3 (20)	**0.03**
Preoperative split renal function [%, mean±SD, (range)]	39.11±12.14 (14-55)	39.27±10.35 (16-52)	0.97
T_1/2_ [minutes, mean±SD, (range)]	30.75±12.18 (18-64)	27.67±7.88 (17-40)	0.38

**Figure 1:**
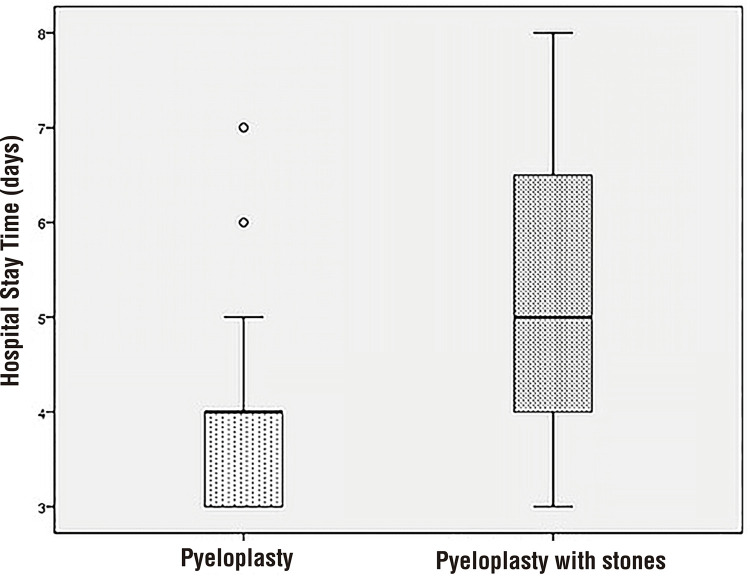
Distribution of postoperative duration of hospital stay between laparoscopic pyeloplasty and laparoscopic pyeloplasty with pyelolithotomy.

**Figure 2:**
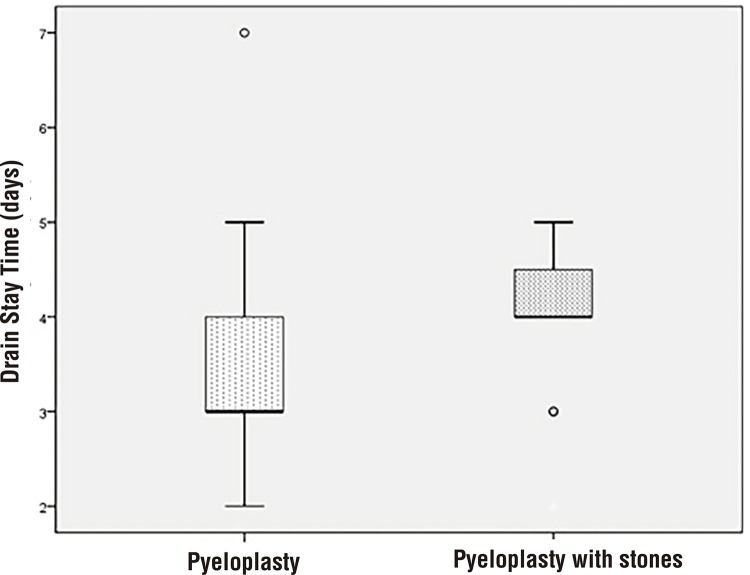
Distribution of time to remove drain between laparoscopic pyeloplasty and laparoscopic pyeloplasty with pyelolithotomy.

The mean operative time was 222.67±65.71 minutes in patients with kidney stones, and in those without renal calculi it was 219.11±75.63 minutes (p=0.88) (Table-2). The mean nephrolithotomy time was 30.67±13.1 minutes in patients with kidney stones. There was no statistically significant difference in anastomosis time between groups (59.64±20.23 vs. 52.33±11.78, p=0.21). There were no perioperative complications, and no patient required conversation to open surgery. The estimated blood loss was similar in both groups (p=0.71) (Table-2). The median hospitalization time and time to remove drain were significantly longer in group-2 than in group-1 (p=0.005 and p=0.005, respectively) (Table-2) (Figures 1 and 2).

**Table 2:** Operative characteristics of patients.

	Group 1 (n=28)	Group 2 (n=15)	p
Mean operative time [minutes, mean±SD, (range)]	219.11±75.63 (110-420)	222.67±65.71 (90-360)	0.88
Anastomosis time [minutes, mean±SD, (range)]	59.64±20.23 (30-120)	52.33±11.78 (30-65)	0.21
Renal Pelvis Reduction [n (%)]	8 (28.6)	3 (20)	0.54
Estimated blood loss [mL, median (IQR), (range)]	50 (38) (10-150)	50 (60) (10-100)	0.71
Drain stay time days, [median (IQR), (range)]	3 (1) (2-7)	4 (1) (2-5)	**0.005**
Hospital stay [days, median (IQR), (range)]	4 (1) (3-7)	5 (3) (3-8)	**0.005**
Stent time [days, mean±SD, (range)]	42.54±12.30 (30-92)	45.80±18.37 (30-103)	0.69
Success [n (%)]	26 (92.9)	14 (93.3)	0.73
Follow-up [months, median (IQR), (range)	12 (26) (3-52)	14 (23) (3-54)	0.66
Postoperative split renal function at 3th months [%, median (IQR), (range)]	41 (17) (14-50)	41 (15) (17-48)	0.97
Postoperative T_1/2_ [minutes, mean±SD, (range)]	16.75±4.53 (8-26)	16.93±2.76 (12-23)	0.89

The median number of stones removed was 1 (range 1-18) and the mean stone size was 13±5.24mm (range, 6-28mm) (Table-3). Most of these stones were located in lower calyces (Table-3). Flexible nephroscope and nitinol basket were used to extract the stones. The stones were collected in specimen retrieval bag and extracted at the end of the surgery.

**Table 3:** Sstone characteristics of patients.

Variables	
Mean stone diameter [mm, mean±SD, (range)]	13.00±5.24 (6-28)
Number of stones removed [median(IQR), (range)]	1 (1) (1-18)
**Stone location [n(%)]**	
Renal pelvis	3 (20)
Middle calyx	3 (20)
Lower calyx	9 (60)
Mean lithotomy time [minutes, mean±SD, (range)]	30.67±13.1 (5-50)
Using flexible nephroscope for lithototmy [n(%)]	12 (80)
Stone free [n(%)]	14 (93.3)

The mean follow-up of patients with and without renal stones was not statistically different between groups (p=0.66) (Table-2). All patients became stone-free at 3 months, and with no evidence of residual stone on imaging after operation. At the 3rd month after operation the success of operation was assessed objectively with diuretic renogram. These diuretic renograms revealed normal drainage in 26/28 patients in group-1 and in 14/15 patients in group-2.

## DISCUSSION

The surgical treatment of kidney stones in the last 25 years has developed from primarily an open surgery to include non-invasive shock wave lithotripsy (SWL) and several minimally invasive treatment modalities. SWL, flexible ureteroscopy, and percutaneous nephrolithotomy (PCNL) have markedly reduced the morbidity of treating kidney stones. Nonetheless, renal stone patients needing simultaneous treatment of UPJ obstruction might still require open procedures, because they cannot be treated with SWL. The success rate of open pyeloplasty and pyelolithotomy has been reported as 90% (7). However, open surgery has several handicaps including unfavorable cosmetic results due to long incision, substantial postoperative pain because of the lombotomy incision, and prolonged hospitalization and convalescence times. Hence, open surgery for renal stones with UPJ obstruction has diminished because the introduction of minimally invasive surgery.

Several minimal endoscopic interventions have been evolved to reduce the unfavorable drawbacks of open pyeloplasty and pyelolithotomy. Until recently, antegrade endopyelotomy with concomitant PCNL was the minimally invasive treatment of choice with success rate of 56-90%, but much lower than open surgery (8-13). However, it is not indicated to perform antegrade endopyelotomy with PCNL in some cases of severe hydronephrosis, crossing vessels, strictures longer than 2cm, renal failure, bleeding disorder, and extended periureteral inflammation (14). In our series, we detected significant crossing vessels with helical CT in 15 patients without renal stones and 3 patients with renal stones. Another significant disadvantage of PCNL performed with antegrade endopyelotomy is requiring an upper pole access which may cause pleural excursion. Also, its lower success rate is another disadvantage in comparison with laparoscopic and robotic surgery (15).

The development of technology allowed for the combination of the benefits of minimally invasive surgery with the higher success rate of open procedures by presenting laparoscopic pyeloplasty and pyelolithotomy (2, 16). The first dismembered laparoscopic pyeloplasty series was presented by Schuessler et al. in 1993 (2), and its success rate was between 96-98% (16-19). In addition, it has been shown that laparoscopic pyeloplasty is an efficient procedure as a salvage operation in cases of failed endopyelotomies (12). The feasibility and safety of laparoscopic pyeloplasty with concomitant pyelolithotomy was supported by several studies that reported excellent stone-free rates and functional results, ranging between 90-100% (Table-4) (4, 20-27). However, these studies included small number of patients because of the rarity of the combination of UPJ obstruction and renal stone. Lusuardi and Janetscheck suggested that laparoscopic pyelolithotomy may be offered as first choice treatment when variations are involved, such as UPJ obstruction (28). Therefore, the European Association of Urology Guidelines recommend laparoscopy for stones with UPJ obstruction (29). However, while laparoscopic pyeloplasty and pyelolithotomy have very important advantages, the major drawbacks of laparoscopic surgery are longer operative time and a steep learning curve (4).

**Table 4:** Contemporary series of pyeloplasty with concomitant pyelolithotomy.

	#renal units (n)	#stones [n (range)]	Stone size (mean mm)	Operative time (minutes)	EBL (mL)	Hospital stay (days)	Pyeloplasty success rate (%)	Stones-free rate (%)	Follow-up (months)
Ramakumar	20	1^*^ (1-28)	N/A	276	145	3.4	90	90	12
Ball	7	2.5 (1-4)	10.3	N/A	N/A	N/A	100	85.7	8.5
Srivastava	20	2^*^ (1-12)	15	168	69.5	4.9	90	100	34
Stein	15	6.2 (1-21)	5.8	174	53.3	1.6	93.3	80	5.4
Rivas	12	N/A	1.53^*^	N/A	N/A	N/A	91.6	100	N/A
Stravodimos	13	8.2 (1.32)	8.7	218.8	N/A	7	100	84.6	30.2
Naitoh	4	1.6 (1-4)	11.5	277	9.5	N/A	N/A	100	N/A
Kouriefs	6	(1-6)	1-20	180 (150-220)^*^	50 (50-100)	2*	100	100	(18-87)
Nambirajan	1	1	2	N/A	N/A	N/A	100	100	N/A
Present study	18	1^*^ (1-18)	13	222.67	50^*^	5^*^	93.3	100	14^*^

^*^: median

This study presents the author’s first experiences with laparoscopic pyeloplasty and pyelolithotomy, which seems to be a safe and efficient procedure for patients presenting with UPJ obstruction and concomitant kidney stones. Our result showed no significant impact of concomitant pyelolithotomy on operative time, estimated blood loss, anastomosis time, complications, and success rates when pyeloplasty was performed simultaneously with pyelolithotomy. Despite of approximately 30 minutes for stone extraction, the operative time of laparoscopic pyeloplasty with pyelolithotomy was similar to laparoscopic pyeloplasty without pyelolithotomy. None of patients required conversion to open surgery. Our conversion rate is similar to other series ranging between 0-5.4 (14, 19). However, the mean hospitalization time was longer in patients with renal stones (p=0.016). The mean hospital stay of our patients is similar to those previously published in the literature (Table-4).

Currently, the general consensus on the follow-up after laparoscopic pyeloplasty is to perform a diuretic renogram at the postoperative 3^rd^ month. An unobstructed drainage in diuretic renogram and/or IVP is accepted as success (30). In our study, diuretic renogram at the postoperative 3rd month demonstrated that T_1/2_ decreased from 23.18 to 15.8 in patients without renal stones (p=0.034), and from 24.55 to 16.45 in patients with renal stones (p=0.038). Moreover, there was not any difference between the groups in relation to the reduction of T_1/2_. Because the most of the failure of pyeloplasty occurs usually within the first postoperative year, and an unobstructed 3-month renogram is followed by unobstructed 1-year renogram, the follow-up of the patients undergoing pyeloplasty is not necessary after the first year (30, 31). However, several studies demonstrated that the long-term success rates of endopyelotomy and pyeloplasty are worse than previously reported (32, 33). Most failures occur within the first 2 years for pyeloplasty and endopyelotomy and there are still failures as follow-up continues (33). In our study, the median follow-up of the patients with and without renal stones were 11 and 12 months, respectively, and these are not different from the other series. Therefore, we still follow-up our patients accordingly with annual ultrasound, although our success rate was over 90% in both groups. These findings indicate the laparoscopic pyeloplasty with concomitant pyelolithotomy is a highly effective procedure for UPJ obstruction and renal stones. Also, the presence of renal stones in hydronephrotic kidney due to the UPJ obstruction does not affect the success and complication rates of laparoscopy.

The stone-free rate of our operations was 93.3%. This rate is similar to the rates of previous studies (Table-4). The most important factors influencing the stone-free rate of these operations are the number, size, and location of stones (24). If the patient has a small single stone in renal pelvis, the rate of being stone-free is higher than the patients with multiple, large calyceal stones. The median number of stones in our patients was 1, and the mean size of these stones was 13mm. These results are in accordance with the results of the studies in the literature (4, 27). However, our stone size is slightly larger than the stones of the other studies (22, 24, 27). Our mean operative times of both types of operations are in accordance with the other results presented in the literature (4, 24).

The limitation of this study is its retrospective nature. However, the number of patients is one of the highest in the literature (Table-4). Another importance of this study is that the data were collected from a single institution.

## CONCLUSIONS

Laparoscopic pyeloplasty with concomitant pyelolithotomy is an effective and safe alternative to open surgery for patients with renal stones and UPJ obstruction. The addition of pyelolithotomy might prolong the operation and hospitalization time. However, it does not affect the success or complication rates.
